# Clinical and Radiological Outcome of Anterior Only Stabilization for AO Type B and C Subaxial Cervical Spine Injury: An Observational Study

**DOI:** 10.31729/jnma.8857

**Published:** 2025-01-31

**Authors:** Ram Sharma Subedi, Bhadra Hamal, Kabita Devi Baral, Badri Rijal, Mahesh Karmacharya, Prem Kumar Sah, Gaurav Raj Dhakal

**Affiliations:** 1 Department of Orthopedics, National Academy of Medical Sciences, Mahaboudha, Kathmandu, Nepal; 2 Bharatpur Hospital, Bharatpur, Chitwan, Nepal

**Keywords:** *anterior*, *disability*, *fusion*, *neurology*

## Abstract

**Introduction:**

AO type B and C subaxial cervical spine injuries are highly unstable and require surgical fixation for the stabilization. This study aims to determine their outcome after anterior stabilization clinically and radiologically.

**Methods:**

This was an observational longitudinal study conducted at tertiary level trauma center, from March 2021 to April 2022 after ethical approval from Institutional Review Board (Reference Number: 665/2077/78). Based on inclusion criteria total sampling was done. Cervical spine injuries AO type B and C operated with anterior cervical stabilization were included. Descriptive statistics were used to analyze data.

**Results:**

Among 21 total cases, 14 (66.67%) were male and 7 (33.33%) were female with the median age of 40 (IQR 32-51) years. Eleven (52.38%) patients sustained AO type B injury and 10 (47.61%) patients sustained AO type C injury. The commonest mode of injury was fall from height 14 (66.66%) followed by RTA 6 (28.57%) and physical assault 1 (4.76%). Postoperatively there was 33% improvement in incomplete neurology by one grade on ASIA neurology. Pain was evaluated using Visual Analogue Score and disability was evaluated using Neck disability Index scoring with the median value of 2 (IQR 0.4-3) and 10 (IQR 3-13) respectively. Radiographic failure was present in 2(9.52%) patients. Forteen (66.66%) patients showed Grade 1 fusion, six (28.57%) showed Grade 2 fusion, and one (4.76%) showed Grade 3 fusion.

**Conclusions:**

Most of the patients experienced mild pain and disability, stable fusion and low rate of radiographic failure with no new neurological deterioration. Almost half of the injuries occurred at the level of C5-C6.

## INTRODUCTION

Cervical spine injuries occur in 2% to 3% of all blunt trauma.^1^ The subaxial cervical spine (C3 to C7) accounts for approximately two-thirds of cervical fractures and three-quarters of dislocations, with more than 50% of injuries located between C5 and C7.^2^ Cervical spine injury AO type B are tension band injuries and type C are translational injuries.^3^ Cervical spine trauma is one of the most common sites of spinal cord injury (SCI).^4^

Anterior and/or posterior operative stabilization should be done for an unstable cervical spine for immediate stability and for maintaining alignment to promote fusion.^5^ Anterior cervical plating is common since there is an easier dissection and the anterior column can be reconstructed directly.^6^ As this study was done in tertiary level trauma center of the country, the outcome information will be helpful to generate our contextual evidence.

Therefore, this research was designed to find clinical and radiological outcome of anterior stabilization of cervical spine.

## METHODS

This was an observational longitudinal study conducted at National Trauma Center, Kathmandu Nepal. This is the only dedicated tertiary level trauma center in Nepal at present. The study was conducted from March 2021 to April 2022 after ethical approval from the Institutional Review Board (Reference number: 665/2077/78). All patients of the age 18 years or more; having undergone an anterior-only cervical fixation procedure for subaxial AO type B and C injuries and providing informed consent were included in the study. Patient undergoing initial posterior fixation or planned circumferential fixation, compressive or brust fractures requiring multi-segment fixation, fractures due to neoplastic or infectious etiology, ankylosing spondylitis or diffuse idiopathic skeletal hyperostosis (DISH) were excluded from the study.

Informed written consent was taken either in Nepali or in English language, in whichever they felt comfortable. Every precaution was employed to maintain privacy of the patient. A detailed proforma of the participants was filled by the researcher. All patients were managed at National Trauma Center with regular follow up at six weeks, three months and six months. Data were collected in the emergency department and Out Patient Department (OPD) in a proforma. The data collected were analyzed in scores. Patients were followed up in the OPDs. Demographic profile of the patient was evaluated by operation records to collect patients' clinical and demographic information including: age, date of injury, gender, diagnosis, date of operation, procedure performed, and mechanism of injury.

Clinical outcome was assessed by using three tools, visual analogue scale (VAS), neck disabilty index (NDI) and American Spinal Injury Association (ASIA) neurology. The visual analog scale (VAS) is a validated, subjective measure for acute and chronic pain. Scores were recorded by making a handwritten mark on a 10cm line that represented a continuum between "no pain" and "worst pain".^7^ NDI is based on validated questionarre with total score of 50 and includes 5 continuum between no disability to complete disability.^8^ The ASIA neurology is a standardized examination consisting of a myotomal-based motor examination, dermatomal based sensory examination, and an anorectal examination. Based on the findings of these examinations, an injury severity or grade and level are assigned.^9^

Radiological outcome was assessed gathering all preoperative and post-operative radiological data for each case from plain radiographs (XR) with cervical spine lateral view in flexion, extension and neutral position at final follow up to see fusion, instability, alignment and subsidence. The postoperative radiograph was taken on the first postoperative day. Follow up radiographs were taken at regular clinical intervals, including six weeks, three months, and six months. The latest follow-up radiograph, which had to be at least 6 months postsurgery was the one used for analysis, except in the case of early radiographic failure that may have been identified as early as 2 weeks after surgery.

Radiographic failure is defined as translation of greater than 3.5 mm and/or a change in angulation of greater than 11° or gross descriptive failure (such as screw breaking or plate dislodgment) in the interval between the immediate postop film and the most recent follow-up radiograph.^5^

Fusion is defined by noting the presence or absence of bridging trabeculae across the interspace, radiolucent lines between the graft and vertebral body, and loss of endplate definition. A fusion grade was assigned using the Bridwell et al. fusion grade defined as grade I (fused with remodeling and trabeculae), grade II (graft intact, not fully remodeled and incorporated, no lucencies), grade III (graft intact with definite lucency at the top or the bottom of the graft), and grade IV (definitely not fused with graft resorption and collapse).^5^

More than 3.5 mm of intersegmental translation (a summation of the displacement observed between vertebra tracing the posterior line on both the flexion and extension view) is considered unstable.

## RESULTS

Among 21 patients operated 14 (66.66%) were male and 7 (33.33%) female with the median age of 40 (IQR 32-51 years) ranging from 18 to 70 years. 11 (52.38%) patients sustained AO type B injury and 10 (47.61%) sustained AO type C injury. The most common mode of injury was fall from height 14 (66.66%) followed by RTA 6 (28.57%) and physical assault 1 (4.76%). There were 1 (4.76%) patient with C3-C4 injury, 3 (14.28%) patients with C4-C5 injury, 11 (52.38%) patients with C5-C6 injury , and 6 (28.57%) with C6-C7 injury.

As measured by VAS score, 12 (57.14%) patients had mild pain, and median VAS score was 2 ( IQR: 0.4-3) Similarly, on the basis of NDI, 10 (47.61%) patients had mild disability and median NDI was 10 (IQR: 3-13), (Table 1).

**Table 1:** VAS score and NDI in patients with type B and C subaxial cervical spine injuries (n=21).

n (%)		
VAS score	No pain	6 (28.57)
	Mild pain	12 (57.71)
	Moderate pain	2 (9.52)
	Severe pain	1 (4.57)
NDI	No disability	7 (33.33)
	Mild disability	10 (47.61)
	Moderate disability	2 (9.52)
	Severe disability	2 (9.52)

Neurologically preoperatively 1 (4.76%) patient had ASIA A neurology, 2 (9.52%) had ASIA B neurology, 4(19.04%) had ASIA C neurlogy, 4 (19.04%) had ASIA D neurology and 10 (47.61%) had ASIA E neurology. Postoperatively 1 (4.76%) patient had ASIA A neurogy , 1 (4.76%) had ASIA B, 2 (9.52%) had ASIA C, 4 (19.04%) had ASIA D and 13 (61.90%) had ASIA E neorology. No new neurogical deterioration was observed.

Radiographic failure, defined as a change in translation of greater than 3.5 mm and a change in angulation greater than 11°, was present in 2 (9.52%) patients, 1 (4.76%) of these two patients who had radiographic failure also had gross migration or pullout of the screws from the vertebral body. Change in angulation greater than 11° and translation greater that 3.5 mm were both present in 1 of the 2 patient (Table 2).

Fusion was assessed using the Bridwell criteria. Of the 21 patients, 14 (66.66%) patients showed Grade 1 fusion, 6 (28.57%) patients showed Grade 2 fusion, and 1 (4.765) patient showed Grade 3 fusion (Table 2).

**Table 2:** Instability and fusion in patient with Type B and C subaxial cervical spine injury (n=21).

Percentage		
Instability	Yes	2 (9.52)
	No	19 (90.47)
Fusion Grade	Grade I	14 (66.66)
	Grade II	6 (28.57)
	Grade III	1 (4.76)
	Gross fusion failure	-

## DISCUSSION

In this study pain was evaluated using an analog pain scale that ranges from 0 (no pain) to 10 (maximal pain). On VAS scoring our median value was 2 (IQR 0.4-3), which could be compared with the studies done by Woodworth et al.^10^ and Kanna et al.^11^ with the mean value of 1.47 with SD 1 and 2.2 with SD 1.2 respectively.

In our study most common mode of trauma was fall injury 14 (66.66%) followed by RTA 6 (28.57%), which was followed by physical assault 1 (4.76%). Which was different from the study done by Anissipour et al.^12^ where he observed motor vehicle injury as a leading cause. Which could be due to different geographics and place of the study being urban areas of developed countries. But was similar to the study done by Madan et al. where he found most common mode of trauma to have been fall from the height (66.7%) followed by roadside accident (33.3%).^13^

On our observation of disability via NDI, 7 (33.33%) had no disability, 10 (47.61%) had mild disability, 2 (9.52%) had moderate disability and 2 (9.52%) had severe disability with median value 10 (IQR 3-13). Which was also comparable to the study done by Madan et al., where they found no disability in 27.3%, mild in 62.6%, moderate in 6% and severe in 4% of study population.^13^ But there were differences from the study done by Gao et al.^2^ Their mean value of 14±8.6 could be attributed to the larger sample size 218 cases in their study in comparison to ours.

A complete neurological deficit by itself does not constitute an indication for surgery, as neuronal death cannot be expected to recover even after surgery.^14^ However the neurologic status is among the most important determinants of surgical treatment and is also the most important prognostic factor.^4^ Neurogically our result showed that there was no change in ASIA A neurology with significant one level improvement in ASIA B, C and D neurology when analysed pre and post operatively. No new neurogical deterioration was observed. This result more or less could be compared with the study conducted by Madan et al., where out of 99 patients, there was no complete neurological compromise but found varrying degree of one level recovery in incomplete neurological injury.^13^

Gao et al. found that, a total of 143 of the original 218 patients (65.61%) and 140 of 191 patients with incomplete paralysis (73.32%) showed varying degrees of neurological function recovery.^2^ In our study no improvement was observed from complete paralysis and only 33.33% improvement observed with incomplete paralysis. The reason could be very short duration of follow up in our study.

All ASIA A patients remained as A, while 46.61% incomplete neurology improved by one grade in study done by Kanna et al.^11^ Which was comparable to our study where we found 33.33% improvement in incomplete neurology by one grade. In the study done by Anissipour et al. comparable findings were observed but 50% improvement in neurology by one level was observed both from complete and incomplete impairment in their study.^12^ This difference might be due to shorter interval between trauma and surgery and longer follow up period than our study.

Radiographic failure in our study was observed in 9.52% of patients. Similarly regarding fusion using the Bridwell criteria, of the 21 patients, 66.66% patients showed Grade 1 fusion, 28.57% showed Grade 2 fusion, and 4.76% showed Grade 3 fusion. No gross fusion failure was observed.

In similar study conducted by Johnson et al, there was 13% radiographic failure with fusion grade I,II,III by 58%, 15%, 27% respectively with 7% cases of gross fusion failure.^5^

Radiographic failure was also comparable to study done by Anissipour et al where they found it to be 8% with mean translation of 0.86±2.24 and mean kyphosis 2.59±5.36.^12^ Endplate and facet fractures were reported as risk factors for ACDF failure.^5^ For us several technical reasons might have contributed to a low rate of treatment failure. Patients undergone ACDF had locking plates. Preferences were given to the long screw fixation. Modern designs of anterior cervical plates with locking screw plate interfaces have led to greater application of anterior fixation to cervical trauma.^12^ Positioning screws within 2 mm of the posterior vertebral cortex would optimize fixation and could decrease failure rates.^12^ Radiographic differences that correlated with surgical revision were the amount of translation post-operatively and its progression, in addition to progressive post-operative segmental kyphosis.^15^

We found the median age of the patients to be 40 (IQR 32-51) years with a range from 18 to 70 years, with 66.66% male and 33.33% female. Similar finding was observed by Gao et al. in which the mean age was 42.6 years , ranging from 21 to 72 years with 55% male and 45% female.^2^ Which was also comparable to other similar study done by Madan et al^13^ and Jack et al.^15^ The comparability to different study signifies the predilection of specific sex and age group to cervical spine trauma. Although different study resulted similar frequency agewise, some variability still existed with the study done by Madan et al.^13^ Which could be because of variability in sample size and geographical differences. Subaxial cervical spine subluxations and dislocations represent a common injury pattern in active age group.^16^ The frequency of AO type B (52.38%) and C (47.61%) that we observed was also found to be similar with the study done by Madan et al 60.6% were type B, 33.3% were type C.^13^

Level of injury we found was comparable to the study done by Kanna et al, with the most common level being C5-C6 (51.20%) followed by C6-C7 (30.80%), C4-C5 (12.80%), C3-C4 (5.10%) respectively.^11^ Similar findings were obtained from the study done by Anissipour et al.^12^ and Jack et al.^15^ The reason behind could be subaxial cervical spine is having considerable mobility and proximity to the more rigid thoracic region.^13^ That's why the same level was found to have been affected with similar percentage of involvement

Advantages of the anterior approach, especially in trauma, include the supine patient position, direct access to herniated discs, and less surgical trauma, blood loss, and wound complications.^6^ Thus, the anterior approach is referred to as the standard approach to the injured subaxial cervical spine in multiple guidelines.^17^

Here we could acknowledge limitations within the study. The small sample size and lack of long term follow-up limited the assessment of the functional status and long term complications. Different patients were operated by different surgeons. The outcome may be dependent upon the surgical expertise of individual surgeon, which wasn't considered in our study.

## CONCLUSIONS

In this study majority of the patients experienced mild pain and disability. Neurological recovery by one grade was observed in one-third of the patients with incomplete injuries, with no new neurological deterioration. Most of the patients had stable fusion and low rate of radiographic failure. There were more middle-aged patients, with almost half of the injuries occurring at the level of C5-C6. The most common mode of injury was fall from height. Further studies with larger sample sizes and extended follow up are needed for long term and better assessment of the outcomes.
